# Supplementary material for: Coincident evolution and functional adaptation of the taxonomically restricted genes ivph-3 and gon-14 in Caenorhabditis nematodes
Source: Biol Open. 2025 Oct 16;14(10):bio062018. doi: 10.1242/bio.062018 (PMC12570147; doi:10.1242/bio.062018)
Supplement: Supplementary information [file biolopen-14-062018-s1.pdf]

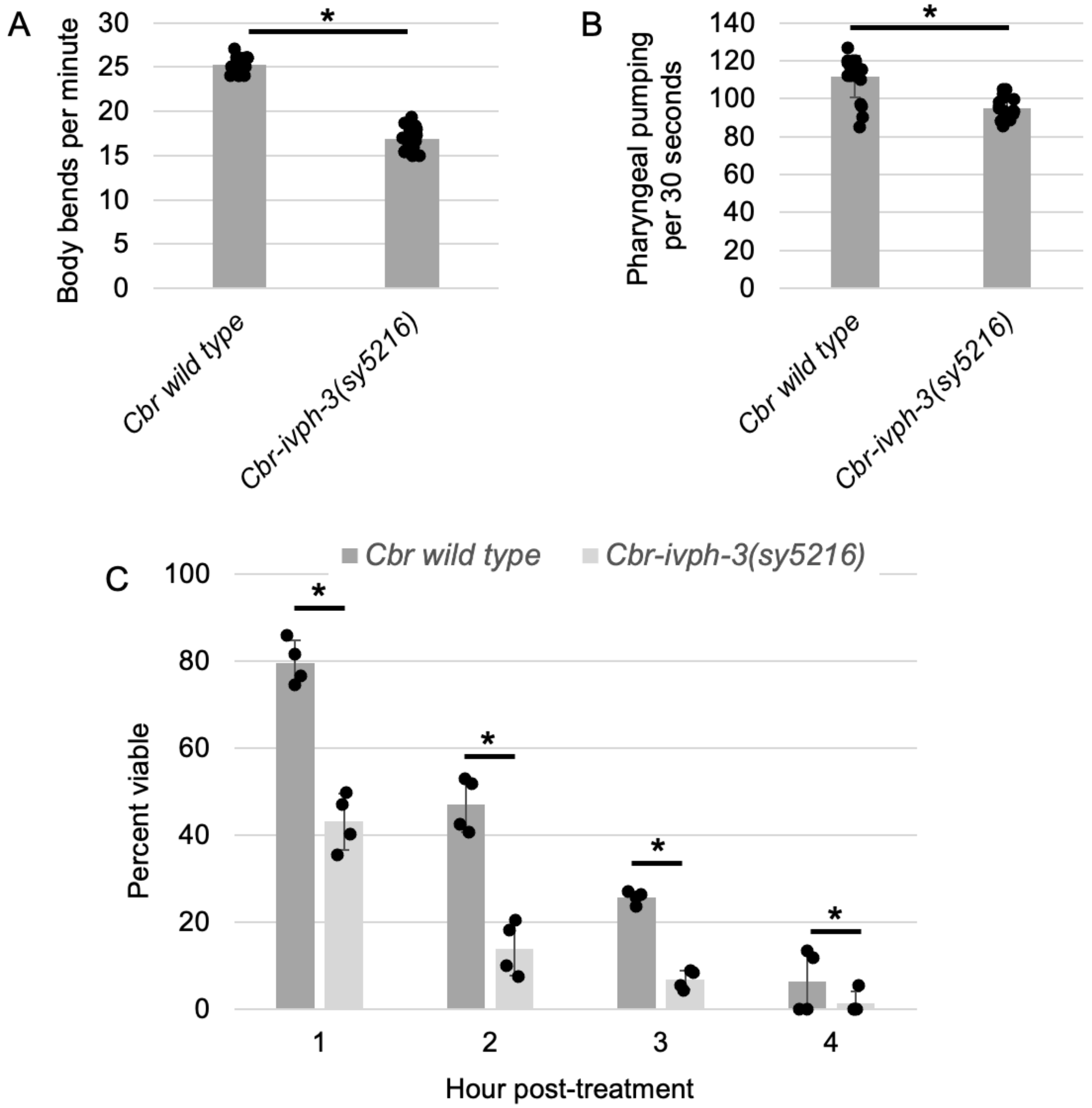

Fig. S1. Phenotypic defects in *Cbr-ivph-3* mutants. A. Adult *Cbr-ivph- 3(sy5216)* mutants exhibit decreased movement compared to wild-type controls. n = 20 animals in a total of two batches for both strains. Bars represent mean ± SD. B. Adult *Cbr-ivph-3(sy5216)* mutants exhibit decreased pharyngeal pumping compared to wild-type controls. n = 20 animals in a total of two batches for both strains. Bars represent mean ± SD. * indicates statistically different (p<0.05, Student's unpaired t-test). C. Adult *Cbr-ivph-3(sy5216*) mutants exhibit increased sensitivity to oxidative stress compared to wild-type controls. Worms were treated with 200mM PQ for 4 hours and survival was measured at one-hour intervals over four hours. n = 300 to 470 in a total of four trials for all strains. Bars represent mean proportion of survival per well ± SD, with proportion from each individual trial indicated. * indicates statistically different (p<0.05, two way ANOVA with Bonferroni correction for multiple comparisons).

Biology Open • Supplementary information

```
                     1 ........10........20........30........40........50........60
Cel-GON-14           1 -------------------DQALTKEFIKSGIPLRAVQDRNLNMLAFNMNSEYSLPTVETM
Cel-IVPH-3           1 PFEHTSEHG-YSSVERYNEEVATRFLLGNGLPLTSIEDHDLRTFLTTLNPSRSLPEPSNM
Cel-LIN-15B          1 ---PTQETAHHLDADPKTTELMARFFISQGIPFECAHEPAFLELMKHVDPNCVIPPTNVT
Cel-F19F10.11        1 --------------------EVLSQFMISQGIPFENIKDSSFKKLLEHFEPGSVVPTVKEL
Cel-ETS-6            1 -FEHLAPGTSDCNSEPTTTDTVCEEFLVSQGIPFESVRDSSLKKLIKHLNPNCEVPTEEEL

                    61 ........70........80........90.......100.......110.......120
Cel-GON-14          43 HSFLEKIADAKA-NAKLNFDDSPVTITFDTTT-------FNNRHYLAFTVHYYHIRSRMR
Cel-IVPH-3          60 TTKA---YGEYRPSLSYQKSIGPINVTIEAIR-------KNDEIFLSISVHYYDSLGERH
Cel-LIN-15B         58 KKLVDKISTSSKPQVNYTKTVGPLSVTIDICG-------DEDEKYLAFSIHYFEDLYERK
Cel-F19F10.11       42 ERSMKFKR---TQKPIDLSIHGPVSITVDLVKHFGD---AEDKMYLVFSVHHFENSGDRT
Cel-ETS-6           60 KCSINKIP---IPKIVNSRRAGPISVTLDVVRAKVSAEEPVKKHYIAFSVHHFENLIQRR

                   121 .......130.......140.......150.......160.......170.......180
Cel-GON-14          95 RCIFLREYDMTGPIVPSVMTAIQKTIEES-GSRLKITTVVAGKAEFED-ALEDMHSFKQV
Cel-IVPH-3         110 NTVHFEKIILADYEGKVVADRLRRVIDANKSMNFGISNILSPNLRMLTLVAENM-PFKNR
Cel-LIN-15B        111 NAIYLRKILLTELDSNSLLTNIRRSVNSYSFSNVKFTNIVCPNEEICKLVEESA-VVKRY
Cel-F19F10.11       96 DAVFLKRVGYSQLNYQTIVMHIRDSMSIHKYKNLSVTNILASNRIIAHRLENDVHSTVNF
Cel-ETS-6          117 DTIFFKKVNEAEISVTMIIAHIQNASRNFKYRDFTITNMVSPDRYIFDFLAERTSQKLNH

                   181 .......190.......200.......210.......220.......230.......240
Cel-GON-14         153 MICFSQNINKFAETLIEHPVFAGPLQKLRDFITKFPTHRNSWGKFKSYI--VKKRTYGEY
Cel-IVPH-3         169 FICFFSYLSNIAREVIQIPEFLSSLKLLREYVGALRKHPEVYTKFRKML--IESKITTDI
Cel-LIN-15B        170 NVCFYNYVTRFVADLMEIEEFSSGLTQLRTFVRYMKQNSDMYSKFRRMQ--LQKNAELDI
Cel-F19F10.11      156 YICFQHHITSFVCALLDMEPFSSRLQNLREFVRKAKSDYKMRSYMHECLNKKKKYSDNSL
Cel-ETS-6          177 YTCTYNRISEFANSVINSPAVRTGLNSLRIFIVSLKIGPTYKLFAKT-L--DAEKINNLK

                   241 .......250.......260.......270.......280.......290.......300
Cel-GON-14         211 PEVD-NGHWITTLDFITKCLHMHRLFSEFVST----SAQLQYIDVEDNVNIVYLHSLLNL
Cel-IVPH-3         227 PALDVESDWLSTLQFLSTCGLLNETFSNLHEN----WCMPKYLDAVQENSMAFLLDFLLV
Cel-LIN-15B        228 PSID-SGDWHSTAIFLTRCLVWHDTFTEFCGK----LDILHYIDNETFNHLIYLQRLLQQ
Cel-F19F10.11      216 PPME-DTSWQSKMNFLKRCLELSSLFLDTVILNYEDFGNATIITSATFIDLKVIFKILQV
Cel-ETS-6          234 LPTE-DGSWVSTYAFLSRCVELHTTLIDYCSTMSGGRKRTQHISNSDFDNLQLADQILRA

                   301 .......310.......320.......330.......340.......350.......360
Cel-GON-14         266 CKSALKEMVDVKATIADVIPAIADISFSLIVTTSKQTVVDAVSTIFNQLLLPFVEQS--D
Cel-IVPH-3         283 LCNVATQICSEDSSVSQVLYSMSIVNNAIE-NCGIVEAREKMRSTFSKYYSSISNGKIGD
Cel-LIN-15B        283 CMKHCRELSIPNNSISQVVPAIMSIRNFIASNSMGYRFQKRIRDSFTTSFKEITSGPSQD
Cel-F19F10.11      275 CSKYSQELSGPDSSVSQVIPAIRSIRSCLVKVTVGKAFGEDVRKCFTDTFQDFTAGPTSM
Cel-ETS-6          293 CVQIVHELSAPNNSISQVIPAIRKIRHVILSDTTKCSGEGRLRKNFTDTFKKVTQGSAST

                   361 .......370.......380.......390.......400.......410.......420
Cel-GON-14         324 IHRFALFLHPL-RHGSDLLASADWMKIKTDLSSHLSLREAAEAT-L-----AHPSSF-DN
Cel-IVPH-3         342 FYSISALLDPRYGYSPMIFSEEDWENVEGKLLKEMSTTHQ-------------------K
Cel-LIN-15B        343 RYDIATLLDPRFAYRDTVYTAQTWRSLEKKVIDDFVNSDLQNDKNFYQDISI--LNQEQR
Cel-F19F10.11      335 KYHIATILDPRFAYCETMYQNETWSAIEGQLRDAFVSKRF-TAKDISQLKYTVPADRESR
Cel-ETS-6          353 RYHLATFLDPRFAYQEDIYPMETWLEIEKELEEQFLIRLQ-TPCDFVQIVEDLPKEYYKR

                   421 .......430.......440.......450.......460.......470.......480
Cel-GON-14         376 TITADKEVNAFSQYVCRVT--GVETGIMDWWARHA-GRFPRLYKYARELFIIPAFSIDAA
Cel-IVPH-3         383 NLIVRKELQRYRELLVNRPLFDESNTPGMWWNDLQ-EELQFMYKKWFEYSGLPAVSIDGK
Cel-LIN-15B        401 YDIIKKEFAYYRQTSFVERP-EENENSNHWWGMRQ-TDMEFLAVIAREYLASPAVSIDAG
Cel-F19F10.11      394 IKIFSSELENYKQILKTSDS-ENFKNPFKFWKVHQRKGMKSLAFLARYYLACPAVAIDAN
Cel-ETS-6          412 VQILQREIEVYKKLIQN----QRARDPFEFWRIHQDDSIIYLSQLAKEFLACPAVSIDAF

                   481 .......490.......500.......510.......520.........
Cel-GON-14         433 YYLGEY-GILTHSINESDI-SKRKMLLRAASELVEYRSKGSLM------
Cel-IVPH-3         442 RFFAKG-GKLAHLFATLDEELHFKAMLL-AQSSQDFVGRGSASILIFNQ
Cel-LIN-15B        459 YYFGNG-GKFQHICHTYSH-QRLENCLALAGNYQTFRGKGASVDVISQ-
Cel-F19F10.11      453 YFVGEGSGSLVRACNMYRDEVELAAYLNAAAVEQQYRGIG---------
Cel-ETS-6          468 QFFGED-GKYNRMCSRYTNDQHLSAYLDVASSFQEYRPHG---------
```

Biology Open • Supplementary information

Fig. S2. Amino acid sequence alignment of the LIN-15B domain from Cel-GON-14, Cel-IVPH-3, Cel-LIN-15B, Cel-F19F10.11, and Cel-ETS-6. Amino acids identical in three or more proteins are shaded black, with conserved amino acids shaded gray. Sequences corresponding to predicted beta sheets in IVPH-3 are underlined with blue arrows, the RNAse H fold alpha helices are underlined with red stripe, and the alpha helices that contribute to a conserved four alpha helix bundle adjacent to the RNAse H fold are underlined with green stripe. LIN-15B, F19F10.11 and ETS-6 also contain one or more THAP or THAP-like domains, but these are separate from the LIN-15B domain (Clouaire *et al.*, 2005).

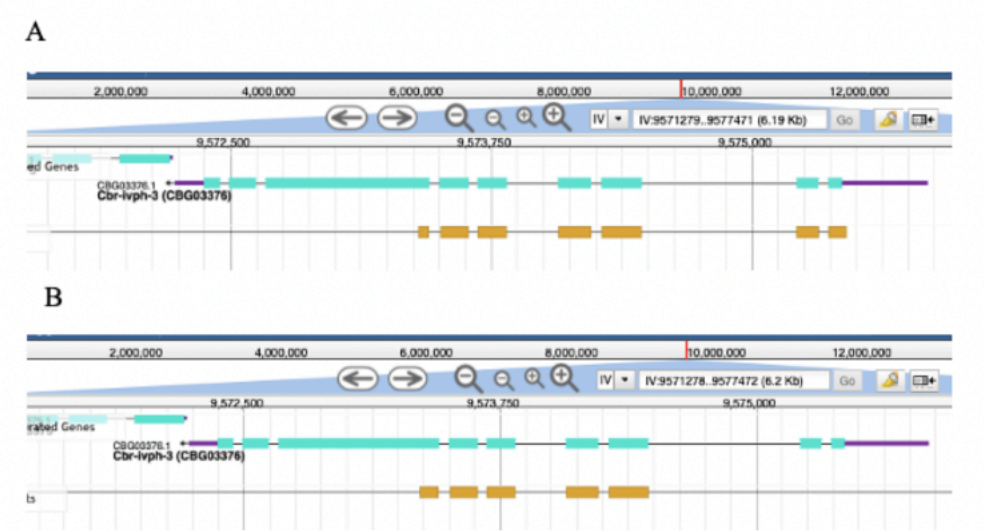

Fig. S3. J browse screen shot of *Cbr-ivph-3* gene model (green) aligned with Blastn sequence matches for two RT-PCR products generated with a *Cbr-ivph-3*-specific primer combined with an SL1-specific primer (yellow). A. A longer product was obtained that corresponds to the predicted gene model. B. A shorter product corresponds to a transcript that excludes annotated exons 1 and 2.

Biology Open • Supplementary information

Table S1. List of DE genes in *Cbr-ivp-3(sy5216)*.

Available for download at

https://journals.biologists.com/bio/article-lookup/doi/10.1242/bio.062018#supplementary-data

Table S2. *Cbr-ivp-3(sy5216)* DE genes with significant changes compared to wild type.

Available for download at
https://journals.biologists.com/bio/article-lookup/doi/10.1242/bio.062018#supplementary-data

Table S3. Overlap of *Cbr-spr-4(gu163), Cbr-htz-1(gu167)* and *Cbr-ivp-3(sy5216)* DE genes

Available for download at
https://journals.biologists.com/bio/article-lookup/doi/10.1242/bio.062018#supplementary-data

Table S4. Overlap between up-regualted DE genes of *Cbr-spr-4(gu163), Cbr-htz-1(gu167)* and *Cbr-ivp-3(sy5216)*

Available for download at
https://journals.biologists.com/bio/article-lookup/doi/10.1242/bio.062018#supplementary-data

Table S5. Overlap between down-regualted DE genes of *Cbr-spr-4(gu163), Cbr-htz-1(gu167)* and *Cbr-ivp-3(sy5216)*

Available for download at
https://journals.biologists.com/bio/article-lookup/doi/10.1242/bio.062018#supplementary-data

Table S6. Nematode strains used in this study

Available for download at
https://journals.biologists.com/bio/article-lookup/doi/10.1242/bio.062018#supplementary-data

Table S7. Details for transgenes used in this study

Available for download at
https://journals.biologists.com/bio/article-lookup/doi/10.1242/bio.062018#supplementary-data

Table S8. Primers used in this study

Available for download at
https://journals.biologists.com/bio/article-lookup/doi/10.1242/bio.062018#supplementary-data

Table S9. Sequences used to produce tree in Fig. 2B

Available for download at
https://journals.biologists.com/bio/article-lookup/doi/10.1242/bio.062018#supplementary-data

Table S10. Sequences used in LIN-15B domain alignment (Fig. S2).

Available for download at
https://journals.biologists.com/bio/article-lookup/doi/10.1242/bio.062018#supplementary-data

Table S11. Steps used in analysis of RNA seq data using the Galaxy platform

Available for download at
https://journals.biologists.com/bio/article-lookup/doi/10.1242/bio.062018#supplementary-data

Table S12. GO term enrichment in *Cbr-spr-4(gu163)* RNA seq data.

Available for download at

https://journals.biologists.com/bio/article-lookup/doi/10.1242/bio.062018#supplementary-data

Table S13. GO term enrichment in *Cbr-htz-1(gu167)* RNA seq data.

Available for download at

https://journals.biologists.com/bio/article-lookup/doi/10.1242/bio.062018#supplementary-data

Table S14. GO term enrichment in *Cbr-ivph-3(sy5216)* RNA seq data.

Available for download at

https://journals.biologists.com/bio/article-lookup/doi/10.1242/bio.062018#supplementary-data

Table S15. GO term enrichment in in the overlap of *Cbr-spr-4(gu163), Cbr-htz-1(gu167)* and *Cbr-ivph-3(sy5216)* RNA seq data.

Available for download at
https://journals.biologists.com/bio/article-lookup/doi/10.1242/bio.062018#supplementary-data

Table S16. Foldseek hit set for Cel-IVPH-3 (AF-Q86DD2-F1-v4) against PDB (human, mouse, zebrafish, Drosophila, Ascaris, Diploscapter)

Available for download at
https://journals.biologists.com/bio/article-lookup/doi/10.1242/bio.062018#supplementary-data

Table S17. Foldseek hit set for Cel-IVPH-3 (AF-Q86DD2-F1-v4) against AFDB50 (human, mouse, zebrafish, Drosophila, Ascaris, Diploscapter)

Available for download at
https://journals.biologists.com/bio/article-lookup/doi/10.1242/bio.062018#supplementary-data

Biology Open • Supplementary information

Table S18. Foldseek hit set for Cel-IVPH-3 (AF-Q86DD2-F1-v4) against AFDB50 (Caenorhabditis)

Available for download at
https://journals.biologists.com/bio/article-lookup/doi/10.1242/bio.062018#supplementary-data

Table S19. Analyses from Large et al. (2025): Jensen-Shannon Distance and Tau

Available for download at
https://journals.biologists.com/bio/article-lookup/doi/10.1242/bio.062018#supplementary-data

Dataset 1.

Available for download at
https://journals.biologists.com/bio/article-lookup/doi/10.1242/bio.062018#supplementary-data

Biology Open • Supplementary information
